# Assessing the State of Knowledge Regarding the Effectiveness of Interventions to Contain Pandemic Influenza Transmission: A Systematic Review and Narrative Synthesis

**DOI:** 10.1371/journal.pone.0168262

**Published:** 2016-12-15

**Authors:** Patrick Saunders-Hastings, Jane Reisman, Daniel Krewski

**Affiliations:** University of Ottawa, McLaughlin Centre for Population Health Risk Assessment, Ottawa, Ontario, Canada; University of Hong Kong, HONG KONG

## Abstract

**Background:**

Influenza pandemics occur when a novel influenza strain, to which humans are immunologically naïve, emerges to cause infection and illness on a global scale. Differences in the viral properties of pandemic strains, relative to seasonal ones, can alter the effectiveness of interventions typically implemented to control seasonal influenza burden. As a result, annual control activities may not be sufficient to contain an influenza pandemic.

**Purpose:**

This study seeks to inform pandemic policy and planning initiatives by reviewing the effectiveness of previous interventions to reduce pandemic influenza transmission and infection. Results will inform the planning and design of more focused in-depth systematic reviews for specific types of interventions, thus providing the most comprehensive and current understanding of the potential for alternative interventions to mitigate the burden of pandemic influenza.

**Methods:**

A systematic review and narrative synthesis of existing systematic reviews and meta-analyses examining intervention effectiveness in containing pandemic influenza transmission was conducted using information collected from five databases (PubMed, Medline, Cochrane, Embase, and Cinahl/EBSCO). Two independent reviewers conducted study screening and quality assessment, extracting data related to intervention impact and effectiveness.

**Results and Discussion:**

Most included reviews were of moderate to high quality. Although the degree of statistical heterogeneity precluded meta-analysis, the present systematic review examines the wide variety of interventions that can impact influenza transmission in different ways. While it appears that pandemic influenza vaccination provides significant protection against infection, there was insufficient evidence to conclude that antiviral prophylaxis, seasonal influenza cross-protection, or a range of non-pharmaceutical strategies would provide appreciable protection when implemented in isolation. It is likely that an optimal intervention strategy will employ a combination of interventions in a layered approach, though more research is needed to substantiate this proposition.

**Trial Registration:**

PROSPERO 42016039803

## 1. Introduction

Each year, influenza infection is responsible for hundreds of thousands of hospitalizations, tens of thousands of deaths, and billions of dollars in healthcare costs and lost productivity in the United States alone [1, 2]. At the same time, there is an ever-present threat of an antigenic shift occurring in the influenza virus, producing a new strain to which humans possess little or no immunity and causing an influenza pandemic with even more catastrophic potential. This has occurred four times in the past hundred years, at unpredictable intervals and with varying degrees of severity. The 1918 Spanish flu remains one of the worst public health catastrophes in recorded human history [3], resulting in between 20 and 50 million deaths globally [4–7].

Key concerns surrounding a future pandemic relate to surges in community illness attack rates and, by extension, hospitalization demand [8–10]. The just-in-time nature of resource delivery in hospitals could make it difficult to adapt to such surges [11, 12]. Taken together, these risks could lead to disruption of health services, compounding the social, economic, and health burdens associated with a pandemic. The inherent uncertainty surrounding such pandemics presents challenges in mounting an appropriate and effective response. Integration of best practices as informed by past influenza pandemics may help in developing effective responses to future pandemics.

This study examines the effectiveness of any intervention to contain human transmission of influenza infection during a future pandemic of unknown severity. To accomplish this, we conducted a systematic review of existing systematic reviews (SR) and meta-analyses (MA) on pandemic influenza interventions. Recognizing that there is substantial variation in where, how, and when interventions are implemented, we sought to better understand the impact of such interventions. Given continuing fears surrounding the threat of avian influenza virus (H5N1 and H7N2) infection in poultry and humans [13, 14], increasing viral diversity of influenza strains circulating in swine populations [15], and escalating human-animal proximity and interaction [16, 17], this article provides timely insight to support future pandemic planning efforts.

## 2. Methods

### 2.1 Overview

The review methodology was developed in keeping with PRISMA [18] guidelines for systematic reviews (S1 Table); a protocol developed *a priori* is published in the National Institute for Health Research International Prospective Register of Systematic Reviews (PROSPERO). Briefly, we conducted a systematic review of existing SRs and MAs dealing with pharmaceutical and non-pharmaceutical interventions to interrupt pandemic influenza transmission and infection. Pharmaceutical interventions include vaccination policies and antiviral use. Non-pharmaceutical interventions include school and work closures, social distancing and contact reduction, use of masks, hand hygiene, and cough etiquette. Where feasible and appropriate, differential effectiveness according to age was noted during data extraction.

### 2.2 Search strategy

Systematic literature searches were conducted on July 5, 2016 using PubMed (all dates), Medline (1946-present), Embase (1947-present), Cochrane Library (all dates) and the Cumulative Index to Nursing and Allied Health (CINAHL; all dates). The general search strategy is presented in Table 1, with database-specific variations documented in the supplemental material (S2 Table).

**Table 1:** Systematic review search strategy as executed in Medline.

1	Influenza, human/
2	Exp Influenzavirus A/
3	1 or 2
4	Pandemics/
5	(pandemic* adj3 (influenza* or flu* or grippe)).tw.
6	4 or 5
7	3 and 6
8	Systematic review.tw.
9	Meta-analysis.tw.
10	Meta analysis.tw.
11	Or/8-10
12	7 and 11

The search excluded research on seasonal influenza and non-influenza disease outbreaks, which would both be of reduced applicability in studying interventions specifically targeting pandemic influenza. The search was conducted on 5 July, 2016, with no language or date restrictions. In cases where a full report was not available, we contacted the authors to request any manuscripts based on the identified abstract. The search was complemented by searching the reference lists of included reviews and *ad hoc* grey literature searches using Google Scholar.

### 2.3 Eligibility criteria and study inclusion

Articles were imported into Endnote X7.5^™^ and were subjected to blind title and abstract appraisal by two independent reviewers. Discrepancies automatically pushed articles to full review. Full texts were sought for articles retained for full review, and again subjected to blind review by two independent reviewers. Conflicts were resolved by consensus and third-party arbitration as necessary. Articles were excluded if they met one of the *a priori* exclusion criteria listed in Table 2. Studies were considered to address an influenza “pandemic” if they assessed an intervention implemented during the first or second wave of a pandemic, after which the annually circulating strain was viewed as a “seasonal” influenza.

**Table 2:** Exclusion criteria for systematic reviews and meta-analyses of pandemic influenza interventions.

Criterion	Rationale
Does not deal with human populations	Animal models may not give accurate representation of impact in humans
Does not include studies on pandemic influenza, but deals exclusively with seasonal influenza or other condition	Experience of pandemic influenza may not reflect that of seasonal influenza
Exclusively reviews in vivo and/or in vitro studies, or mathematical modeling studies	Purpose of study is to examine the behaviour of influenza within human populations, rather than genetic considerations
Does not review an intervention to contain pandemic influenza infection	Purpose of this review is to quantify intervention effectiveness
Does not use infection/transmission risk/rate as an outcome measure	Purpose of this review is to quantify intervention potential to contain pandemic transmission
Only the abstract is available	Must be able to assess article in its entirety
Not a peer-reviewed systematic review or meta-analysis article	Seeking to compare over-arching intervention patterns across heterogeneous settings

### 2.4 Data extraction and analysis

Data from retained articles were extracted to a piloted Excel spreadsheet by two independent reviewers. Spreadsheet categories offer information pertaining to the study populations, interventions, and outcomes. The principal summary measures of relative intervention effect are risk and odds ratios. Methodological heterogeneity of the included systematic reviews—particularly with respect to research questions, inclusion criteria, intervention specifics, and outcome measures—precluded pooling of data for a new meta-analysis, as well as the use of funnel plots to assess the potential for publication bias. Instead, a narrative synthesis is presented for each of the interventions evaluated in a past review, highlighting current knowledge and unfilled data gaps.

### 2.5 Quality assessment

The quality of articles retained for data extraction was assessed by two independent reviewers using the AMSTAR tool (S3 Table). The 11-item questionnaire was developed for application across a broad range of public health interventions [19, 20] and has been widely applied over the past decade [21, 22], including for reviews of seasonal influenza interventions [23–25]. An SR can achieve a maximum score of 10, and an MA a maximum score of 11. Following the approach set out in past publications [21, 23], reviews receiving a score of 9–11 were classified as high quality, 5–8 as moderate-quality, and 0–4 as low-quality. Inter-reviewer disagreements regarding scoring were resolved by consensus only when they resulted in differential quality categorization (low, moderate, high). Although review quality was not used as an exclusion criterion, the level of evidence was noted and integrated into a discussion of results and formulation of conclusions.

## 3. Results

**Fig 1:**
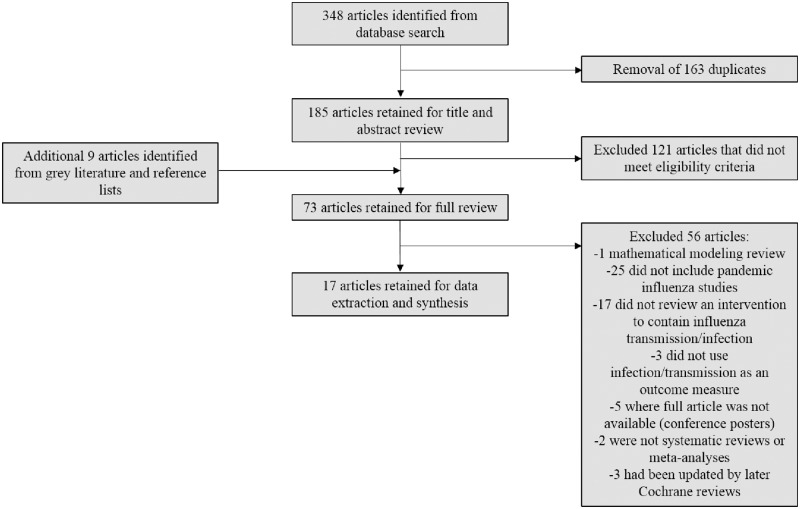
Systematic review flow diagram.

A total of 348 citations were retrieved from the execution of the search strategy discussed. Following the removal of duplicates, 185 articles were subject to title and abstract review, with 64 retained for full review. An additional 9 articles were identified from searches of reference lists and the grey literature; all were reviewed in full. Of these 73 articles, 17 were selected for quality assessment and data extraction. Fig 1 summarizes the study selection process; articles omitted during full review are summarized in S4 Table, along with the reason for their omission.

### 3.1. Included reviews

In total, 17 reviews were retained, covering six types of intervention to prevent pandemic influenza infection. Eight [26–33] review the effectiveness of pandemic influenza vaccine in preventing influenza and influenza-like illness (ILI); three [34–36] examine the impact of antivirals; two [32, 37] review the effectiveness of seasonal influenza vaccines in preventing pandemic influenza infection; two evaluate the impact of personal protective measures (hand-washing, mask use) [38, 39]; one [40] analyzes the impact of school closure; and another [41] reviews the efficacy of traditional Chinese medicine (TCM). One review [42] evaluates the economic viability of a wide range of pharmaceutical and non-pharmaceutical measures, concluding that social distancing, antiviral prophylaxis, school closure, and vaccination are likely to be cost-effective in all settings, while quarantine is never cost-effective. Across these reviews, 33 meta-analyses of intervention impact were conducted. The characteristics of individual reviews are summarized in Table 3, with results and associated implications for intervention impact described in subsequent intervention-specific subsections. Tables summarizing the results of the quantitative analyses performed in the included reviews are available in the appendices for pandemic vaccination (S5 Table), antiviral prophylaxis and treatment (S6 Table), seasonal influenza vaccination (S7 Table), and personal protective measures (S8 Table). Results from the reviews on school closure and TCM are not reported in Tables, as only a single review was available for each.

**Table 3:** Summary of reviews included in the systematic review of pandemic influenza interventions.

Systematic Review	Population	Total Studies (N)	Pandemic Studies for Meta-analysis	Pandemic Meta-analysis Population Size (N)	Intervention	Outcome	AMSTAR Quality (low, moderate, high)	Quality of Evidence*
Breteler et al., 2013	Schoolchildren in China during 2009 pandemic	41	1	95,244	Vaccination (two doses of PANFLU1)	Laboratory-confirmed influenza	High	Only a single study was retrieved
Chien et al., 2010	Civilian and military populations during 1918 pandemic	13	13	1,956,492	Mixed killed bacterial vaccines	Influenza incidence	Moderate	Significant heterogeneity among studies; low scientific quality of 1918 vaccine studies; inconsistent reporting of influenza incidence
Demicheli et al., 2014	Healthy adults and pregnant women during 1968 pandemic	90	6	33,768	1968 and 2009 pandemic vaccines	Influenza or ILI cases	High	Methodological quality was rated as good for 10%; high risk of bias for 20%; impact of bias could not be determined for 70%
Fielding et al., 2014	General population during 2009 pandemic	11	11	1,527	Oseltamivir	Duration of viral shedding	Moderate	Significant heterogeneity noted; prevented meta-analysis and limits scope
Jackson et al., 2013	General population during 1918, 1968, and 2009 pandemics	79	57	N/A	School closure	Cumulative and peak influenza attack rates	Moderate	Significant heterogeneity noted; prevented meta-analysis and limits scope
Jefferson et al., 2008	General population during 1968 pandemic	22	10	12,575	Amantadine prophylaxis	Influenza or ILI cases	High	Significant heterogeneity noted; little information on randomization procedures for studies reviewed
Jefferson et al., 2014	Healthy children (under 16) during 2009 pandemic	75	5	Not reported	2009 pandemic vaccine	Influenza infection	High	Generally poor methodological quality of studies included; poor reporting and high risk of bias
Li et al., 2016	General population during 2009 pandemic	30	12	1,469	Traditional Chinese medicine	Duration of viral shedding	High	Small sample size limits statistical power
Li et al., 2015	General population during 2009 pandemic	28	28	135,347	Seasonal influenza vaccine	Pandemic influenza infection	High	12 of 28 studies had high risk of bias; significant heterogeneity noted among case-control studies
Manzoli et al., 2011	General population during 2009 pandemic	33	18	18,444	2009 pandemic vaccine	Influenza seroconversion	Moderate	Most studies included were sponsored by companies developing the vaccine under study
Mizumoto et al., 2013	General population during 2009 pandemic	17	8	Not reported	Mass antiviral prophylaxis and contact tracing	Secondary infection risk	Moderate	Heterogeneous, arbitrary definitions of "contact", case ascertainment, study setting, and treatment duration
Mukerji et al., 2015	General population during 2009 pandemic	7	3	Not reported	N-95 masks	Economic benefit	High	Results are of limited utility; limited inclusion of clinical data to inform effectiveness estimates
Osterholm et al., 2012	Canadian and European general population during 2009 pandemic	5	5	Not reported	2009 pandemic vaccine	Laboratory-confirmed influenza	Moderate	All studies were observational and of low statistical power
Perez Velasco et al., 2012	General population during 2009 pandemic	44	44	Not reported	Any	Cost-effectiveness, utility, or benefit	High	Evidence is of low quality and generally inconclusive; variations in intervention implementation
Wong et al., 2014	General population during 2009 pandemic	10	1	149	Hand hygiene and facemask	Laboratory-confirmed influenza or ILI	High	Small sample size of included trial lead to significant imprecision and limited generalizability
Yin et al., 2012	General population during 2009 pandemic	27	27	3,011,641	Seasonal and pandemic influenza vaccines	Laboratory-confirmed influenza	High	Most studies included were of low or moderate quality; significant heterogeneity noted
Yin et al., 2011	General population during 2009 pandemic	16	16	17,921	Pandemic influenza vaccine	Influenza seroconversion	High	Nine of 16 studies were of low quality; significant heterogeneity noted

* Quality of studies is reported as indicated by the quality assessment of the original authors.

### 3.2 Quality assessment

Inter-reviewer agreement on the quality of the systematic reviews assessed was strong. Of the 17 reviews collected, 10 were rated as being of high methodological quality; 6 were of moderate quality; and one was of low quality (S9 Table). It should be noted, however, that the quality of the systematic reviews alone does not suggest that the conclusions drawn can be viewed with a high degree of certainty. Rather, insufficient data, appreciable heterogeneity, and wide confidence intervals were noted across many of the reviews. Comments on the quality of evidence obtained from the review, as well as an independent assessment of the methodological rigor of that review, are also included in Table 3.

### 3.3. Pandemic influenza vaccine effectiveness

Of the eight reviews assessing the effectiveness of pandemic influenza vaccines, seven report on the effectiveness of the 2009 pH1N1 vaccine [26, 28–33], one reports on the 1968 pandemic vaccine [28], and one reports on the efficacy of killed bacterial vaccines used during the 1918 pandemic [27]. With few exceptions—notably the 1918 bacterial vaccines, used prior to identification of the influenza virus—there appears to be a general consensus that pandemic vaccines were effective across age groups in preventing pandemic influenza infection.

With regard to the 2009 H1N1 pandemic, Breteler *et al*. [26] report on a study of schoolchildren in China [43], where a vaccine effectiveness of 87% (95% CI: 75%-93%) was found. The review by Demicheli *et al*. [28] included a single pandemic study [44], which estimated a risk ratio of 0.11 (95% CI 0.06–0.21) associated with the 2009 inactivated pandemic vaccine in pregnant Japanese women. A 2014 review by Jefferson *et al*. [29] did not pool the results of five pandemic vaccination studies [45–49], but reported a consensus across studies that pandemic vaccines provided a significant protective effect against infection, with vaccine efficiency ranging from 71.9%-96%. Similarly, Osterholm *et al*. [31] did not pool the results of five observational studies of the effectiveness of monovalent pandemic H1N1 vaccination, but reported a median effectiveness of 69% (range: 60%-93%). Yin *et al*. [32] examined 11 case-control studies reporting on pandemic influenza vaccination and laboratory-confirmed influenza, calculating a combined odds ratio of 0.14 (95% CI 0.07–0.27).

Both Manzoli *et al*. [30] and Yin *et al*. [33] reviewed the seroprotective effect of different H1N1 pandemic vaccines. Manzoli *et al*. [30] found a significant impact of higher vaccine concentrations for single-dose vaccines (RR of seroconversion 1.05; 95% CI 1.03–1.07 per dose step increase), but no significant effects were associated with higher concentrations in two-dose vaccines, administration of a second dose (except in children), or addition of vaccine adjuvants such as aluminum. Yin *et al*. [33] did not report quantitative effects on infection, but concluded that pandemic vaccination significantly impacted seroconversion, regardless of administration of one or two doses or the addition of aluminum hydroxide as an adjuvant, neither of which significantly improved the immune response.

Demicheli *et al*. [28] reported that all forms of the 1968 inactivated vaccine were effective in preventing both confirmed influenza and ILI. While the effect was greater for influenza than ILI, it should be noted that all results for confirmed influenza derived from a single study [50]. The authors found no significant effect of live aerosol vaccines. Lastly, Chien *et al*. [27] evaluated the effectiveness of mixed killed bacterial vaccines in reducing influenza incidence during the 1918 pandemic, finding no significant protective effect; this is not surprising given that bacterial vaccines are likely to be ineffective against viral pathogens.

### 3.4 Antiviral effectiveness

Three systematic reviews evaluated studies on the role of antiviral prophylaxis and treatment in reducing pandemic influenza infection [34–36]. Fielding *et al*. [34] found that oseltamivir treatment received within 48 hours of symptom onset tended to reduce the duration of viral shedding (3–5 days) relative to no treatment (4–9 days) and to treatment received over 48 hours after onset (5–7 days). This contraction of the infectious period of the index case could reduce secondary infections [34]. Mizumoto *et al*. [36] reported that secondary infection rates generally decreased in situations where mass oseltamivir prophylaxis had been employed, with a median secondary infection risk of 2.1% (relative to 16.6% among those not receiving prophylaxis). In both cases differences in study design, exposure, and treatment strategies precluded pooled estimates of effectiveness. Jefferson *et al*. [35] evaluated the effectiveness of amantadine prophylaxis during the 1968 influenza pandemic, reporting significant protective effects against confirmed influenza (RR 0.27; 95% CI 0.17–0.46) and ILI (RR: 0.78; 95% CI 0.74–0.83). However, the authors point out that amantadine significantly increased adverse gastrointestinal and nervous system effects, suggesting that they should only be used in emergency situations, and may not be appropriate for mass prophylaxis.

### 3.5 Seasonal influenza vaccine effectiveness

Two systematic reviews, both from the 2009 pandemic, report on the cross-protection of seasonal influenza vaccines against pandemic influenza infection for the three Northern hemisphere influenza seasons between 2007 and 2010 and the two Southern hemisphere influenza seasons between 2008 and 2009 [32, 37]. Li *et al*. [37] report a non-significant risk increase across four randomized control trials (RR: 1.13; 95% CI 0.56–2.29), though we argue that these findings should be interpreted with caution, due to small sample size (n = 1,515). They also report a non-significant protective effect across 16 case-control studies (n = 40,868, OR: 0.80; 95% CI 0.61–1.05). Yin *et al*. [32] report a similar, non-significant protective effect across 11 case-control studies (n = 31,699, OR: 0.81; 95% CI 0.58–1.13), but found a significant effect when five studies with a high risk of bias were excluded (n = 28,292; OR: 0.66; 95% CI 0.48–0.91). Taken together, these two reviews suggest that seasonal influenza vaccination had a moderate, though non-significant effect in protecting from influenza infection during the 2009 pandemic.

### 3.6 Personal protective measure effectiveness

Of the two systematic reviews analyzing personal protective measures an influenza epidemic, one [39] reported on its effectiveness in preventing infection, while the other [38] discussed its economic benefit. Wong *et al*. reviewed ten studies of hand hygiene and facemask use in developed countries, and obtained an insignificant estimate of risk reduction associated with hand hygiene alone (RR: 0.82; 95% CI 0.66–1.02) but a significant risk reduction when hand hygiene was practiced in conjunction with facemask use (RR: 0.73; 95% CI 0.53–0.99). However, only one of these ten studies [51] was performed in a pandemic setting, with the other nine dealing instead with seasonal influenza control. With small sample size limiting generalizability (n = 149), insignificant risk reductions associated with hand hygiene and facemask use for laboratory-confirmed influenza (RR: 0.64; 95% CI 0.32–1.29) and influenza-like illness (RR: 0.52; 95% CI 0.21–1.29) were found in the pandemic study [51]. Mukerji *et al*. [38] do not report quantitative data on the effectiveness of interventions in preventing infection, but reviewed past cost-effectiveness studies of mask use. Noting important limitations in the studies reviewed, these authors suggest that masks and respirators may be cost-effective, though there is insufficient data to inform more specific interventions.

### 3.7 School closure effectiveness

A single systematic review [40] assessed the impact of school closure across 57 pandemic studies from the 1918, 1968, and 2009 pandemics. Despite reporting a contact rate reduction of 30%-78% in school-aged children, statistical and methodological differences precluded the authors from pooling data for meta-analysis, comparing of optimal intervention strategies, or commenting on statistical significance.

### 3.8 Traditional chinese medicine effectiveness

A review by Li *et al*. [41] examined the effect of Chinese medicines, herbs, extracts, or other ingredients in reducing the duration of viral shedding in individuals infected with pandemic H1N1, both alone and in combination with oseltamivir treatment. In a meta-analysis of 12 studies (n = 1,469), using oseltamivir treatment as a control, the mean duration of viral shedding did not differ significantly between the TCM and oseltamivir treatment groups (mean difference 0.07 days; 95% CI -0.07–0.21). However, a significant reduction in duration of viral shedding was noted in a comparison between a group receiving both TCM and oseltamivir and an oseltamivir control (mean difference −0.52 days; 95% CI −0.96–−0.09).

## 4. Discussion

The present systematic review is the first assess the state of knowledge regarding interventions to prevent pandemic influenza transmission as reported in existing systematic reviews and meta-analyses. This is an important information gap, as the high degree of uncertainty and heterogeneity regarding pandemic outbreaks and response suggests value in analyzing overarching trends in intervention effectiveness. Variability in pandemic environments, including the degree of infectiousness, population demographics and susceptibility, and intervention strategies and timing, inhibit the generalizability of effectiveness measures reported from a small number of studies to other settings and future pandemics.

Some authors [35, 52] have proposed that intervention effectiveness can be expected to mirror what is observed during seasonal influenza epidemics. This viewpoint is problematic for several reasons. First, seasonal influenza epidemics tend not to be considered as emergency situations, and extreme response measures are not employed [42]. This limits the ability to evaluate the effectiveness of interventions such as school closure, facemask use, or quarantine of infected individuals, which would be inappropriate during standard seasonal influenza seasons. As a consequence, there is no conclusive evidence on the impact of these strategies: there was, in fact, substantial uncertainty about which measures to implement during the 2009 pandemic [53, 54]. Second, the assertion that seasonal influenza research remains relevant to pandemic influenza situations remains controversial [55]. Some suggest that intervention effectiveness may increase in pandemic situations, due to media attention and public anxiety increasing rates of adherence [56]; this was the case during the SARS epidemic [53, 57]. Additionally, the uncertain timing of pandemic influenza outbreaks, relative to usual influenza seasons, may alter non-pharmaceutical intervention effectiveness by impacting the relative importance of different modes of transmission, which have been suggested to vary with ambient temperature and relative humidity [39, 58]. In short, there is a need for more targeted reviews examining the empirical data from past pandemic events, where high viral loads, transmission rates, and public anxiety [55, 59] may have impacted the effectiveness of interventions that were implemented.

The results of this review were insufficient to draw concrete conclusions on the effectiveness of most interventions. Of the 17 reviews included, only seven specifically reviewed pandemic influenza situations, while the other ten conducted subgroup analyses: two of these [26, 39] found only a single pandemic study that met their inclusion criteria. The most commonly investigated intervention was pandemic influenza vaccination, which was found to be highly effective in preventing pandemic influenza infection and ILI. This is not surprising, as the 2009 pandemic vaccine was a very close match with the circulating strain [31]. Rather, the concern with pandemic vaccines is that they may not be available in time for the early stages of a pandemic, as vaccine production, development and distribution can take over six months [60, 61]. The few reviews of the interventions that may be employed in the interim reported mixed results. Where measures of statistical significance were reported, only antiviral prophylaxis with amantadine, a drug with known adverse side effects, demonstrated a significant protective effect. A lack of primary data precluded reporting of statistical significance for non-pharmaceutical measures such as hand hygiene, facemask use, and school closure. It is likely that the most impactful and cost-effective approach to interrupting pandemic influenza transmission involves a layered approach combining multiple pharmaceutical and non-pharmaceutical intervention strategies, although this notion is not well explored in the quantitative analysis of included reviews. The overall lack of quantitative primary data on intervention effectiveness supports the crucial role of mathematical modelling in charting pandemic transmission dynamics and supporting the assessment of public health interventions under conditions of uncertainty.

Though not a focus of this article, several reviews were noted that dealt with the effectiveness of treatment options for pandemic influenza [62–70]. While these were beyond the scope of the present review, assessments of four major treatment strategies were found. Results suggest that early treatment with neuraminidase inhibitors can reduce hospitalization [69], ventilator support [71], and death [63–65]. Two reviews [66, 68]—based on a single study—mention a benefit of convalescent plasma for treating severe pandemic influenza cases. Three reviews [62, 66, 68] conclude that there is insufficient evidence to comment on the potential benefit of extracorporeal membrane oxygenation to treat influenza-associated respiratory failure. Two reviews [66, 68] found no benefit of corticosteroid therapy to treat acute lung injury, while another [70] found that it significantly increases nosocomial infection and mortality.

The executed search strategy found no systematic reviews relating to either border control measures or hospital triage protocols. Additional searches of the primary literature suggested a low efficacy associated with border control measures. These include the use of non-contact infrared thermometers in airports to detect infected passengers, where studies from the 2009 pandemic found that the positive predictive value ranged from 0.9% to 76.0%, and was likely to be too low to effectively detect and contain pandemic infection [72, 73]. A study of entry screening for pandemic H1N1 at Auckland International Airport, which focused on encouraging infection reporting and did not use thermal scanning or active screening, reported a screening sensitivity of 5.8%, which the authors concluded to be insufficient to delay the spread of pandemic influenza [74]. The general consensus appears to be that even rigorous and expensive border control measures are unlikely to delay the spread of pandemic influenza by more than a few days [75, 76]. No empirical studies were found that quantified the effectiveness of alternate models of care—such as hospital triage protocols—in containing pandemic influenza.

This present systematic review is subject to certain limitations. First, a decision was made to review existing systematic reviews and meta-analyses, rather than primary literature. This was done in an effort to account for the clinical, methodological, and statistical heterogeneity in this field, while summarizing and assessing current, high-quality research regarding preventative interventions for pandemic influenza, and is consistent with past health intervention research methodologies [77]. While it is possible that this approach omits some primary research, this was deemed unlikely to substantially affect results, given the broad search and inclusion criteria and considering that the last pandemic occurred seven years ago, meaning that recent reviews are likely to have captured all relevant primary literature. This approach provided an efficient means of summarizing and assessing the results of numerous reviews in a single study, allowing a more fulsome discussion of the quality of existing evidence on pandemic influenza interventions than would have been feasible from a review of the primary literature. Second, as high heterogeneity both within and between included studies prevented further meta-analysis, we were necessarily restricted to a narrative synthesis of current research and persisting knowledge gaps. The potential for publication biases was noted, as the marginally protective role of interventions such as hand hygiene and mask use may have been overestimated by the disproportionate publication of significant results (the association was still not found to be significant, however). Location bias was also present, as most results included in the reviews were from higher-income countries, and some interventions, such as mass antiviral prophylaxis, may not be feasible in low-resource settings. Another limitation of this review was that most of the available data were obtained from studies of the relatively mild 2009 H1N1 pandemic; this precluded analysis of the how intervention effectiveness is affected by disease characteristics, and may limit to generalizability of findings to future pandemics of unknown severity. Lastly, outcome reporting bias may have influenced the results, given the variability of influenza case definitions that were used in the primary studies, sometimes with little clinical basis.

## 5. Conclusion

This systematic review provides the first synthesis of existing systematic reviews and meta-analyses on interventions to prevent pandemic influenza infection, comparing findings to advance knowledge and understanding of optimal intervention strategies. Important knowledge gaps persist in this area, particularly with regard to the effect of non-pharmaceutical interventions in limiting transmission and infection. While pandemic vaccination appears to be effective in preventing influenza, it is crucial to prepare for the early phases of a pandemic where vaccines may be unavailable. Future work could focus on the impact of personal protective measures in reducing transmission rates; an important avenue for primary research is the prospective study of intervention effectiveness in infectious disease emergency situations. In the meantime, it is hoped the results of the present review will be of value in informing the development of future pandemic intervention strategies.

## Supporting Information

S1 Table PRISMA Checklist. (PDF)

S2 Table Database-specific search strategies. (PDF)

S3 Table AMSTAR Screening Tool. (PDF)

S4 Table Articles Excluded During Full Review. (PDF)

S5 Table Results of Pandemic Vaccination Analyses Reporting Relative Effects. (PDF)

S6 Table Results of Antiviral Analyses Reporting Relative Effects. (PDF)

S7 Table Results of Seasonal Vaccination Analyses Reporting Relative Effects. (PDF)

S8 Table Results of Personal Protective Measure Analyses Reporting Relative Effects. (PDF)

S9 Table AMSTAR Scoring of Included Studies. (PDF)
