# Supplementary material for: Assessing the State of Knowledge Regarding the Effectiveness of Interventions to Contain Pandemic Influenza Transmission: A Systematic Review and Narrative Synthesis
Source: PLoS One. 2016 Dec 15;11(12):e0168262. doi: 10.1371/journal.pone.0168262 (PMC5158032; doi:10.1371/journal.pone.0168262)
Supplement: S2 Table — (PDF) [file pone.0168262.s002.pdf]

S2 Table. Database-specific search strategies

1. **Medline (OVID)**
    1. Influenza, human/
    2. Exp Influenzavirus A/
    3. 1 or 2
    4. Pandemics/
    5. (pandemic* adj3 (influenza* or flu* or grippe)).tw.
    6. 4 or 5
    7. 3 and 6
    8. Systematic review.tw.
    9. Meta-analysis.tw.
    10. Meta analysis.tw.
    11. Or/8-10
    12. 7 and 11

2. **Embase**
    1. Exp influenza/
    2. Exp influenza A virs/
    3. 1 or 2
    4. Pandemic/
    5. (pandemic* adj3 (influenza* or flu* or grippe)).tw.
    6. 4 or 5
    7. 3 and 6

8. Systematic review.tw.
9. Meta-analysis.tw.
10. Meta analysis.tw.
11. Or/8-10
12. 7 and 11

3. PubMed

1. Influenza, human [MeSH Terms]
2. Influenzavirus A [MeSH Terms]
3. 1 or 2
4. Pandemic [MeSH Terms]
5. Pandemic* [Text Word]
6. [Text Word] (influenza* or flu* or grippe)
7. 5 and 6
8. 4 or 7
9. 3 and 8
10. [Text Word] (systematic review or meta-analysis or meta analysis)
11. 9 and 10

4. Cochrane (Wiley)

1. MeSH descriptor: [Influenza, Human] explode all trees
2. MeSH descriptor: [Influenzavirus A] explode all trees
3. 1 or 2
4. MeSH descriptor: [Pandemics] explode all trees
5. pandemic* (Word variations have been searched)

6. influenza* (Word variations have been searched)
7. flu* (Word variations have been searched)
8. grippe (Word variations have been searched)
9. 6 or 7 or 8
10. 5 and 9
11. 4 or 10
12. 3 and 11
13. Systematic review (Word variations have been searched)
14. Meta-analysis (Word variations have been searched)
15. Meta analysis (Word variations have been searched)
16. Or/13-15
17. 12 and 16

5. CINAHL

1. (MH “Influenza+”)
2. (MH “Pandemic+”)
3. TX (influenza* or flu* or grippe) and TX pandemic*
4. 2 or 3
5. 1 and 4
6. TX (systematic review or meta-analysis or meta analysis)
7. 5 and 6
