# Supplementary material for: Assessing the State of Knowledge Regarding the Effectiveness of Interventions to Contain Pandemic Influenza Transmission: A Systematic Review and Narrative Synthesis
Source: PLoS One. 2016 Dec 15;11(12):e0168262. doi: 10.1371/journal.pone.0168262 (PMC5158032; doi:10.1371/journal.pone.0168262)
Supplement: S3 Table — (PDF) [file pone.0168262.s003.pdf]

S3 Table. AMSTAR Screening Tool

Reprinted from Shea, *et al*. (Development of AMSTAR: A measurement tool to assess the methodological quality of systematic reviews. *BMC Medical Research Methodology* **2007**, *7*, 10) under a CC BY license, with permission from BioMed Central, original copyright 2007.

Item Number	Evaluation Criteria	Scoring
1	Was an 'a priori' design provided? (1)The research question/aim and (2) inclusion criteria should be established before the conduct of the review.	• Yes (1 point)- Must satisfy all five criteria<br>• No (0 points)<br>• Can't Answer (0 points)<br>• Not Applicable (0 points)
2	Was there duplicate study selection and data extraction? (1)There should be at least two independent data extractors and a (2) consensus procedure for disagreements should be in place.	• Yes (1 point)- Must satisfy all five criteria<br>• No (0 points)<br>• Can't Answer (0 points)<br>• Not Applicable (0 points)
3	Was a comprehensive literature search performed? (1) At least two databases must be searched; (2) The report must include years and databases used; (3) keywords and/or MESH terms must be stated; (4) search strategy must be provided where feasible; (5) All searches should be supplemented by consulting current contents, reviews, textbooks, specialized registers, or experts in the field, and by reviewing the references in the studies found.	• Yes (1 point)- Must satisfy all five criteria<br>• No (0 points)<br>• Can't Answer (0 points)<br>• Not Applicable (0 points)
4	Was the status of publication (i.e. grey literature) used as an inclusion criteria? (1) Authors should state they searched reports regardless of publication type; (2) Authors should state whether or not they excluded any reports (from the systematic review) based on their publication status, languages, etc.	• Yes (1 point)<br>• No (0 points)<br>• Can't Answer (0 points)
5	Was a list of studies (included and excluded) provided? A list of (1) included and (2) excluded studies should be provided.	• Yes (1 point)<br>• No (0 points)

	Note: excluded studies can be provided in an appendix or external link	• Can't Answer (0 points)
6	Were the characteristics of the included studies provided? In an aggregated form such as a table, data from the original studies should be provided on the participants, interventions and outcomes. Ranges of characteristics (age, sex, relevant socioeconomic data, disease status, duration , severity, or other diseases should be reported)	• Yes (1 point)<br>• No (0 points)<br>• Can't Answer (0 points)<br>• Not Applicable (0 points)
7	Was the scientific quality of the included studies assessed and documented? A priori methods of assessment should be provided Note: (for example, did they mention an instrument/tool to assess quality?)	• Yes (1 point)<br>• No (0 points)<br>• Can't Answer (0 points)<br>• Not applicable (0 points)
8	Was the scientific quality of the included studies used appropriately in formulating conclusions? Results of the methodological rigor and scientific quality should be considered in the analysis and the conclusions of the review, and explicitly stated in formulating recommendations.	• Yes (1 point)<br>• No (0 points)<br>• Can't Answer (0 points)<br>• Not applicable (0 points)
9	Were the methods used to combine the findings of studies appropriate? For pooled results, a test should be done to ensure studies were comparable, to assess their homogeneity (i.e. Chi-squared test for homogeneity, I2). If heterogeneity exists, a random effects model should be used and/or the clinical appropriateness of combining should be taken into consideration (i.e. is it sensible to combine?)	• Yes (1 point)<br>• No (0 points)<br>• Can't Answer (0 points)<br>• Not applicable (0 points)
10	Was the likelihood of publication bias assessed? Assessment of publication bias should include a combination of graphical aids (e.g. full plots, other available tests) and/or statistical tests (e.g. Egger regression test)	• Yes (1 point)<br>• No (0 points)<br>• Can't Answer (0 points)<br>• Not applicable (0 points)
11	Was the conflict of interest included? Potential sources of support should be clearly acknowledged in both the systematic review and the included studies	• Yes (1 point)<br>• No (0 points)<br>• Can't Answer (0 points)<br>• Not applicable (0 points)
