# Supplementary material for: Assessing the State of Knowledge Regarding the Effectiveness of Interventions to Contain Pandemic Influenza Transmission: A Systematic Review and Narrative Synthesis
Source: PLoS One. 2016 Dec 15;11(12):e0168262. doi: 10.1371/journal.pone.0168262 (PMC5158032; doi:10.1371/journal.pone.0168262)
Supplement: S4 Table — (PDF) [file pone.0168262.s004.pdf]

S4 Table. Articles Excluded During Full Review

Reference	Reason for Exclusion
Aledort, J.E.; Lurie, N.; Wasserman, J.; Bozzette, S.A. Non-pharmaceutical public health interventions for pandemic influenza: An evaluation of the evidence base. *BMC public health* **2007**, *7*, (15 August 2007).	Not a systematic review; result of expert panel in light of dearth of relevant literature
Alshabani, K.; Haq, A.; Miyakawa, R.; Soubani, A. Invasive pulmonary aspergillosis following influenza: A systematic review of literature. *American Journal of Respiratory and Critical Care Medicine. Conference: American Thoracic Society International Conference, ATS* **2014**, *189*.	Conference abstract: authors contacted with no response
Alves Galvão Márcia, G.; Rocha Crispino Santos Marilene, A.; Alves da Cunha Antonio, J.L. Amantadine and rimantadine for influenza a in children and the elderly. In *Cochrane Database of Systematic Reviews*, John Wiley & Sons, Ltd: 2014.	Does not include any studies from a pandemic influenza setting
Aoyagi, Y.; Beck, C.R.; Dingwall, R.; Nguyen-Van-Tam, J.S. Healthcare workers' willingness to work during an influenza pandemic: A systematic review and meta-analysis. *Influenza and other respiratory viruses* **2015**, *9*, 120-130.	Does not deal with interventions to prevent influenza infection; deals with willingness of healthcare workers to attend work
Atashili, J.; Kalilani, L.; Adimora, A.A. Efficacy and clinical effectiveness of influenza vaccines in hiv-infected individuals: A meta-analysis. *BMC infectious diseases* **2006**, *6*, 138.	Does not include any studies from a pandemic influenza setting
Babin, S.M.; Hsieh, Y.H.; Rothman, R.E.; Gaydos, C.A. A meta-analysis of point-of-care laboratory tests in the diagnosis of novel 2009 swine-lineage pandemic influenza a (h1n1). *Diagn. Microbiol. Infect. Dis.* **2011**, *69*, 410-418.	Does not deal with preventing influenza infection; deals with point-of-care laboratory test diagnosis
Baca-Muro, V.I.; Soria-Cedillo, I.F.; Olvera, K.; Garcia-Contreras, F. Prevention of an influenza pandemic in mexico: Establishing a cost-effective alternative for elderly population. *Value in Health* **2009**, *12 (7)*, A425.	Conference poster abstract; authors contacted with no response
Balasingam, S.; Wilder-Smith, A. Randomized controlled trials using human challenge studies for influenza: A systematic review. *Tropical Medicine and International Health* **2015**, *20*, 134.	Does not include any studies from a pandemic influenza setting
Beck, C.R.; Sokal, R.; Arunachalam, N.; Puleston, R.; Cichowska, A.; Kessel, A.; Zambon, M.; Nguyen-Van-Tam, J.S.; Group, U.K.A.E.R. Neuraminidase inhibitors	Does not include any studies from a pandemic influenza setting

for influenza: A review and public health perspective in the aftermath of the 2009 pandemic. *Influenza & Other Respiratory Viruses* **2013**, *7 Suppl 1*, 14-24.	
Bin-Reza, F.; Lopez Chavarrias, V.; Nicoll, A.; Chamberland, M.E. The use of masks and respirators to prevent transmission of influenza: A systematic review of the scientific evidence. *Influenza & Other Respiratory Viruses* **2012**, *6*, 257-267.	Does not include any studies from a pandemic influenza setting
Bozat-Emre, S.; Casaclang, N.; Sinnock, H.C.; Ye, X.; Mahmud, S.M. Effectiveness of seasonal influenza vaccines against influenza a (h1n1) infection in post-pandemic seasons: A systematic review. *Pharmacoepidemiol. Drug Saf.* **2015**, *24*, 178-179.	Conference poster abstract related to post-pandemic seasons
Bozat-Emre, S.; Ye, X.; Morrow, A.; Casaclang, N.; Mahmud, S.M. Effectiveness of the 2009 pandemic h1n1 influenza vaccines in preventing h1n1 infection: A meta-analysis. *Pharmacoepidemiol. Drug Saf.* **2014**, *23*, 161-162.	Conference poster abstract; authors contacted with no response
Brien, S.; Kwong, J.C.; Buckeridge, D.L. The determinants of 2009 pandemic a/h1n1 influenza vaccination: A systematic review. *Vaccine* **2012**, *30*, 1255-1264.	Does not use infection or transmission as outcome measure; reports on predictors of vaccination uptake
Burch, J.; Corbett, M.; Stock, C.; Nicholson, K.; Elliot, A.J.; Duffy, S.; Westwood, M.; Palmer, S.; Stewart, L. Prescription of anti-influenza drugs for healthy adults: A systematic review and meta-analysis. *The Lancet Infectious Diseases* **2009**, *9*, 537-545.	Does not include any studies from a pandemic influenza setting (examines treatment of seasonal influenza)
Cates Christopher, J.; Rowe Brian, H. Vaccines for preventing influenza in people with asthma. In *Cochrane Database of Systematic Reviews*, John Wiley & Sons, Ltd: 2013.	Does not include any studies from a pandemic influenza setting
Cowling, B.J.; Zhou, Y.; Ip, D.K.; Leung, G.M.; Aiello, A.E. Face masks to prevent transmission of influenza virus: A systematic review. *Epidemiology & Infection* **2010**, *138*, 449-456.	Does not include any studies from a pandemic influenza setting
De La Parte, B.; Jimenez, V.; Jesus, M.; Reza, M.; Pardillos Ferrer, L.; Mateos Rodriguez, A.; Montarelo, A. Should asthmatic people recive influenza vaccine and pandemic influenza a (h1n1) vaccine? *Allergy: European Journal of Allergy and Clinical Immunology* **2010**, *65*, 717.	Not a systematic review
Devnani, M. Factors associated with the willingness of health care personnel to work during an influenza public health emergency: An integrative review. *Prehospital and disaster medicine* **2012**, *27*, 551-566.	Does not deal with preventing influenza infection; deals with willingness of healthcare workers to work

Eliakim-Raz, N.; Vinograd, I.; Zalmanovici Trestioreanu, A.; Leibovici, L.; Paul, M. Influenza vaccines in immunosuppressed adults with cancer. In *Cochrane Database of Systematic Reviews*, John Wiley & Sons, Ltd: 2013.	Does not include any studies from a pandemic influenza setting
Gadre, S.; Duggal, A. Use of rescue therapies during the h1n1 pandemic: A systematic review exploring global differences in the management of severe acute respiratory distress syndrome. *Chest. Conference: CHEST* **2015**, *148*.	Conference poster abstract and does not deal with preventing influenza infection
Jagannath Vanitha, A.; Asokan, G.V.; Fedorowicz, Z.; Lee Tim, W.R. Neuraminidase inhibitors for the treatment of influenza infection in people with cystic fibrosis. In *Cochrane Database of Systematic Reviews*, John Wiley & Sons, Ltd: 2016.	No studies were found in this review and does not deal with preventing influenza infection
Jefferson, T.; Demicheli, V.; Rivetti, D.; Jones, M.; Di Pietrantonj, C.; Rivetti, A. Antivirals for influenza in healthy adults: Systematic review. *Lancet* **2006**, *367*, 303-313.	Updated in Jefferson et al., 2008
Jefferson, T.; Del Mar, C.; Dooley, L.; Ferroni, E.; Al-Ansary, L.A.; Bawazeer, G.A.; van Driel, M.L.; Nair, S.; Foxlee, R.; Rivetti, A. Physical interventions to interrupt or reduce the spread of respiratory viruses. *The Cochrane database of systematic reviews* **2010a**, Cd006207.	Updated in Jefferson et al., 2011
Jefferson, T.; Di Pietrantonj, C.; Al-Ansary Lubna, A.; Ferroni, E.; Thorning, S.; Thomas Roger, E. Vaccines for preventing influenza in the elderly. In *Cochrane Database of Systematic Reviews*, John Wiley & Sons, Ltd: 2010b.	Does not include studies on pandemic influenza
Jefferson, T.; Jones, M.; Doshi, P.; Del Mar, C.; Dooley, L.; Foxlee, R. Neuraminidase inhibitors for preventing and treating influenza in healthy adults: A cochrane review. *Health Technology Assessment* **2010c**, *14*, 355-458 104p.	Does not include studies on pandemic influenza
Jefferson, T.; Del Mar, C.B.; Dooley, L.; Ferroni, E.; Al-Ansary, L.A.; Bawazeer, G.A.; van Driel, M.L.; Nair, S.; Jones, M.A.; Thorning, S., *et al.* Physical interventions to interrupt or reduce the spread of respiratory viruses. *The Cochrane database of systematic reviews* **2011**, Cd006207	Did not have sufficient data to carry out subgroup analysis for pandemic influenza outbreaks.
Jefferson, T.; Rivetti, A.; Di Pietrantonj, C.; Demicheli, V.; Ferroni, E. Vaccines for preventing influenza in healthy children. *The Cochrane database of systematic reviews* **2012**, *8*, CD004879.	Updated in Jefferson et al., 2014c
Jefferson, T.; Jones, M.A.; Doshi, P.; Del Mar, C.B.; Hama, R.; Thompson, M.J.; Spencer, E.A.; Onakpoya, I.; Mahtani, K.R.; Nunan, D., *et al.* Neuraminidase inhibitors for	Did not have sufficient data to carry out subgroup

preventing and treating influenza in healthy adults and children. *Cochrane Database of Systematic Reviews* **2014b**, N.PAG-N.PAG 1p.	analysis for pandemic influenza outbreaks.
Jiang, L.; Deng, L.; Wu, T. Chinese medicinal herbs for influenza. In *Cochrane Database of Systematic Reviews*, John Wiley & Sons, Ltd: 2013	Does not include any studies from a pandemic influenza setting
Khazeni, N.; Bravata, D.M.; Holty, J.E.; Uyeki, T.M.; Stave, C.D.; Gould, M.K. Systematic review: Safety and efficacy of extended-duration antiviral chemoprophylaxis against pandemic and seasonal influenza. *Annals of internal medicine* **2009**, *151*, 464-473.	Does not include any studies from a pandemic influenza setting
Lau, L.L.; Nishiura, H.; Kelly, H.; Ip, D.K.; Leung, G.M.; Cowling, B.J. Household transmission of 2009 pandemic influenza a (h1n1): A systematic review and meta-analysis. *Epidemiology (Cambridge, Mass.)* **2012**, *23*, 531-542.	Does not review an intervention; surveys household secondary infection risk
Luke, T.C.; Kilbane, E.M.; Jackson, J.L.; Hoffman, S.L. Meta-analysis: Convalescent blood products for spanish influenza pneumonia: A future h5n1 treatment? *Annals of internal medicine* **2006**, *145*, 599-609 511p.	Does not deal with preventing influenza infection; deals with use of blood products to prevent influenza during Spanish flu
Manzoli, L.; Salanti, G.; De Vito, C.; Boccia, A.; Ioannidis, J.P.; Villari, P. Immunogenicity and adverse events of avian influenza a h5n1 vaccine in healthy adults: Multiple-treatments meta-analysis. *The Lancet Infectious Diseases* **2009**, *9*, 482-492.	Does not include any studies from a pandemic influenza setting
Martin Arias, L.H.; Sanz, R.; Sainz, M.; Treceno, C.; Carvajal, A. Guillain-barre syndrome and influenza vaccines: A meta-analysis. *Vaccine* **2015**, *33*, 3773-3778.	Does not deal with preventing influenza infection; deals with association between vaccination and Guillain-Barré syndrome
Mateus, A.L.; Otete, H.E.; Beck, C.R.; Dolan, G.P.; Nguyen-Van-Tam, J.S. Effectiveness of travel restrictions in the rapid containment of human influenza: A systematic review. *Bulletin of the World Health Organization* **2014**, *92*, 868-880D.	Only review mathematical modeling studies
Mathie Robert, T.; Frye, J.; Fisher, P. Homeopathic oscillococcinum® for preventing and treating influenza and influenza-like illness. In *Cochrane Database of Systematic Reviews*, John Wiley & Sons, Ltd: 2015.	Does not include any studies from a pandemic influenza setting
McMillan, M.; Kralik, D.; Porritt, K.; Marshall, H. Influenza vaccination during pregnancy: A systematic review of effectiveness and safety. *JBI Database of Systematic*	Does not include any studies from a pandemic influenza setting

Reviews & Implementation Reports **2014**, *12*, 281-381 101p.	
Mitchell, M.D.; Mikkelsen, M.E.; Umscheid, C.A.; Lee, I.; Fuchs, B.D.; Halpern, S.D. A systematic review to inform institutional decisions about the use of extracorporeal membrane oxygenation during the h1n1 influenza pandemic. *Critical Care Medicine* **2010**, *38*, 1398-1404.	Does not deal with preventing influenza infection; deals with extracorporeal membrane oxygenation as a rescue therapy
Morton, M.J.; Jeng, K.; Beard, R.; Dugas, A.; Pines, J.M.; Rothman, R.E. Systematic review of interventions to mitigate the effect of emergency department crowding in the event of a respiratory disease outbreak. *Academic Emergency Medicine* **2012**, *19*, S64-S65.	Conference poster abstract that does not deal with preventing influenza infection
Muthuri, S.G.; Myles, P.R.; Venkatesan, S.; Leonardi-Bee, J.; Nguyen-Van-Tam, J.S. Impact of neuraminidase inhibitor treatment on outcomes of public health importance during the 2009-2010 influenza a(h1n1) pandemic: A systematic review and meta-analysis in hospitalized patients. *Journal of Infectious Diseases* **2013**, *207*, 553-563.	Does not deal with preventing influenza infection
Muthuri, S.G.; Venkatesan, S.; Myles, P.R.; Leonardi-Bee, J.; Al Khuwaitir, T.S.; Al Mamun, A.; Anovadiya, A.P.; Azziz-Baumgartner, E.; Baez, C.; Bassetti, M., *et al.* Effectiveness of neuraminidase inhibitors in reducing mortality in patients admitted to hospital with influenza a h1n1pdm09 virus infection: A meta-analysis of individual participant data. *The Lancet Respiratory Medicine* **2014**, *2*, 395-404	Does not deal with preventing influenza infection
Muthuri, S.G.; Venkatesan, S.; Myles, P.R.; Leonardi-Bee, J.; Lim, W.S.; Al Mamun, A.; Anovadiya, A.P.; Araujo, W.N.; Azziz-Baumgartner, E.; Baez, C., *et al.* Impact of neuraminidase inhibitors on influenza a(h1n1)pdm09-related pneumonia: An individual participant data meta-analysis. *Influenza and other respiratory viruses* **2016**, *10*, 192-204.	Does not deal with preventing influenza infection
Ortiz, J.R.; Rudd, K.E.; Clark, D.V.; Jacob, S.T.; West, T.E. Clinical research during a public health emergency: A systematic review of severe pandemic influenza management. *Critical Care Medicine* **2013**, *41*, 1345-1352.	Does not deal with preventing influenza infection
Ott, J.J.; Klein Breteler, J.; Tam, J.S.; Hutubessy, R.C.; Jit, M.; de Boer, M.R. Influenza vaccines in low and middle income countries: A systematic review of economic evaluations. *Human Vaccines Immunotherapy.* **2013**, *9*, 1500-1511.	Does not include any studies from a pandemic influenza setting

Pfister, R.; Kochanek, M.; Leygeber, T.; Brun-Buisson, C.; Cuquemelle, E.; Machado, M.B.P.; Piacentini, E.; Hammond, N.E.; Ingram, P.R.; Michels, G. Procalcitonin for diagnosis of bacterial pneumonia in critically ill patients during 2009 h1n1 influenza pandemic: A prospective cohort study, systematic review and individual patient data meta-analysis. *Crit. Care* **2014**, *18 (2) (no pagination)*.	Does not deal with preventing influenza infection
Prieto-Lara, E.; Llanos-Mendez, A. Safety and immunogenicity of prepandemic h5n1 influenza vaccines: A systematic review of the literature. *Vaccine* **2010**, *28*, 4328-4334.	Does not deal with preventing influenza infection
Rodrigo, C.; Leonardi-Bee, J.; Nguyen-Van-Tam, J.S.; Lim, W.S. Effect of corticosteroid therapy on influenza-related mortality: A systematic review and meta-analysis. *Journal of Infectious Diseases* **2015**, *212*, 183-194.	Does not deal with preventing influenza infection
Rudd, K.E.; Ortiz, J.R.; Clark, D.V.; Jacob, S.T.; West, T.E. A systematic review of clinical interventions for patients with severe pandemic influenza a (h1n1) virus infection. *American Journal of Respiratory and Critical Care Medicine. Conference: American Thoracic Society International Conference, ATS* **2012**, *185*.	Does not deal with preventing influenza infection
Shun-Shin, M.; Thompson, M.; Heneghan, C.; Perera, R.; Harnden, A.; Mant, D. Neuraminidase inhibitors for treatment and prophylaxis of influenza in children: Systematic review and meta-analysis of randomised controlled trials. *BMJ* **2009**, *339*, b3172.	Does not include any studies from a pandemic influenza setting
Smith, S.M.; Sonego, S.; Wallen, G.R.; Waterer, G.; Cheng, A.C.; Thompson, P. Use of non-pharmaceutical interventions to reduce the transmission of influenza in adults: A systematic review. *Respirology* **2015**, *20*, 896-903.	Does not include any studies from a pandemic influenza setting
Thomas Roger, E.; Jefferson, T.; Lasserson Toby, J. Influenza vaccination for healthcare workers who care for people aged 60 or older living in long-term care institutions. In *Cochrane Database of Systematic Reviews*, John Wiley & Sons, Ltd: 2016; Vol. 6.	Does not include any studies from a pandemic influenza setting
Thomas Roger, E.; Lorenzetti Diane, L. Interventions to increase influenza vaccination rates of those 60 years and older in the community. In *Cochrane Database of Systematic Reviews*, John Wiley & Sons, Ltd: 2014.	Does not deal with preventing influenza infection; deals with increasing seasonal vaccine uptake
Thorlund, K.; Awad, T.; Boivin, G.; Thabane, L. Systematic review of influenza resistance to the neuraminidase inhibitors. *BMC infectious diseases* **2011**, *11*, 134.	Does not deal with preventing influenza infection

Venkatesan, S.; Myles, P.R.; Leonardi-Bee, J.; Nguyen-Van-Tam, J.S. Impact of outpatient neuraminidase inhibitor treatment on hospitalisation in patients infected with influenza a (h1n1)pdm09: An ipd analysis. *International Journal of Infectious Diseases* **2016**, *45*, 248.	Does not deal with preventing influenza infection
Wang, K.; Shun-Shin, M.; Gill, P.; Perera, R.; Harnden, A. Neuraminidase inhibitors for preventing and treating influenza in children (published trials only). In *Cochrane Database of Systematic Reviews*, John Wiley & Sons, Ltd: 2012.	Does not include any studies from a pandemic influenza setting
Yang, J.W.; Fan, L.C.; Miao, X.Y.; Mao, B.; Li, M.H.; Lu, H.W.; Liang, S.; Xu, J.F. Corticosteroids for the treatment of human infection with influenza virus: A systematic review and meta-analysis. *Clinical Microbiology and Infection* **2015**, *21*, 956-963.	Does not deal with preventing influenza infection
