# Supplementary material for: Assessing the State of Knowledge Regarding the Effectiveness of Interventions to Contain Pandemic Influenza Transmission: A Systematic Review and Narrative Synthesis
Source: PLoS One. 2016 Dec 15;11(12):e0168262. doi: 10.1371/journal.pone.0168262 (PMC5158032; doi:10.1371/journal.pone.0168262)
Supplement: S5 Table — (PDF) [file pone.0168262.s005.pdf]

S5 Table. Results of Pandemic Vaccination Analyses Reporting Relative Effects

Pandemic	Study	N Studies	Population Size (N)	Vaccine Type	Outcome	Risk Control Group	Risk With Intervention	Relative Effect (95% CI)
2009 H1N1	Breteler et al., 2013	1	269,128	Two doses of PANFLU1	Laboratory-confirmed influenza	687/244,091 (2.8/1,000)	9/25,037 (0.35/1,000)	RR*: 0.127 (0.066-0.246)
2009 H1N1	Demicheli et al., 2014	1	7,328	2009 pandemic inactivated vaccine	ILI**	50/2,407 (20.8/1,000)	11/4,921 (2.2/1,000)	RR: 0.11 (0.06-0.21)
2009 H1N1	Manzoli et al., 2011	18	18,444	One dose non-adjuvanted pandemic vaccine	Influenza seroconversion	Not reported	Not reported	RR: 1.05 (1.03-1.07)
2009 H1N1	Manzoli et al., 2011	18	18,444	Two doses non-adjuvanted pandemic vaccine	Influenza seroconversion	Not reported	Not reported	RR: 1.01 (1.00-1.03)
2009 H1N1	Manzoli et al., 2011	18	18,444	One dose adjuvanted pandemic vaccine	Influenza seroconversion	Not reported	Not reported	RR: 1.10 (1.04-1.17)
2009 H1N1	Manzoli et al., 2011	18	18,444	Two dose adjuvanted pandemic vaccine	Influenza seroconversion	Not reported	Not reported	RR: 1.05 (1.02-1.07)
2009 H1N1	Yin et al., 2012	11	283,826	A(H1N1)pdm09 vaccine	Laboratory-confirmed influenza	6,229/258,154 (24.1/1,000)	71/25,708 (2.8/1,000)	OR= 0.14 (0.07-0.27)

1968 H2N2	Dimecheli et al., 2014	3	3,065	1968 inactivated polyvalent parenteral vaccine	ILI	127/1,377 (92.2/1,000)	149/1,688 (88.2/1,000)	RR: 0.71 (0.57-0.88)
1968 H2N2	Dimecheli et al., 2014	1	2072	1968 inactivated polyvalent parenteral vaccine	Influenza	32/1,042 (30.7/1,000)	15/1,030 (14.7/1,000)	RR: 0.47 (0.26-0.87)
1968 H2N2	Dimecheli et al., 2014	4	4580	Inactivated monovalent parenteral vaccine	ILI	169/1,790 (94.4/1,000)	107/2,790 (38.4/1,000)	RR: 0.35 (0.25-0.48)
1968 H2N2	Dimecheli et al., 2014	1	1923	Inactivated monovalent parenteral vaccine	Influenza	32/1,042 (30.7/1,000)	2/881 (2.3/1,000)	RR: 0.07 (0.02-0.31)
1968 H2N2	Dimecheli et al., 2014	2	1000	Inactivated polyvalent aerosol vaccine	ILI	86/335 (256.7/1,000)	117/665 (175.9/1,000)	RR: 0.66 (0.46-0.95)
1968 H2N2	Dimecheli et al., 2014	2	1009	Inactivated monovalent aerosol vaccine	ILI	86/335 (256.7/1,000)	103/674 (152.8/1,000)	RR: 0.54 (0.32-0.91)
1968 H2N2	Dimecheli et al., 2014	1	19887	Live aerosol vaccine	Influenza	1429/9942 (143.7/1,000)	1,407/9,945 (141.5/1,000)	RR: 0.98 (0.92-1.05)
1918 H1N1	Chien et al., 2010	12	Not reported	Mixed killed bacterial vaccines	Influenza incidence	166,870/1,723,172 (96.84/1,000)	20,087/233,320 (86.09/1,000)	RR: 0.89 (Not reported)

*RR = relative risk

**ILI = influenza-like illness (based on reporting of symptoms rather than clinical diagnosis)
