# Supplementary material for: Assessing the State of Knowledge Regarding the Effectiveness of Interventions to Contain Pandemic Influenza Transmission: A Systematic Review and Narrative Synthesis
Source: PLoS One. 2016 Dec 15;11(12):e0168262. doi: 10.1371/journal.pone.0168262 (PMC5158032; doi:10.1371/journal.pone.0168262)
Supplement: S6 Table — (PDF) [file pone.0168262.s006.pdf]

S6 Table. Results of Antiviral Analyses Reporting Relative Effects

Pandemic	Study	N Studies	Population Size (N)	Antiviral Type	Outcome	Risk Control Group	Risk With Intervention	Relative Effect (95% CI)
2009 H1N1	Fielding et al., 2014	19	1527	Oseltamivir treatment within 48 hours of symptom onset	Duration of viral shedding	No treatment: 4-9 days	3-5 days	Not reported
2009 H1N1	Fielding et al., 2014	11	1527	Oseltamivir treatment within 48 hours of symptom onset	Duration of viral shedding	Treatment >48 hours after symptom onset: 5-7 days	3-5 days	Not reported
2009 H1N1	Mizumoto et al., 2013	8	Not reported	Oseltamivir mass prophylaxis	Secondary infection risk	Median SIR 16.6% (quartile 8.4% -32.4%)	Median SIR 2.1% (quartile 0%-12.2%)	Not reported
1968 H2N2	Jefferson et al., 2008	3	613	Amantadine prophylaxis	Influenza cases	62/307 (202.0/1,000)	17/306 (55.6/1,000)	0.27 (0.17-0.46)
1968 H2N2	Jefferson et al., 2008	6	11,962	Amantadine prophylaxis	ILI	1,682/5,298 (317.5/1,000)	1,647/6,664 (247.2/1,000)	0.78 (0.74-0.83)
