# Supplementary material for: Assessing the State of Knowledge Regarding the Effectiveness of Interventions to Contain Pandemic Influenza Transmission: A Systematic Review and Narrative Synthesis
Source: PLoS One. 2016 Dec 15;11(12):e0168262. doi: 10.1371/journal.pone.0168262 (PMC5158032; doi:10.1371/journal.pone.0168262)
Supplement: S7 Table — (PDF) [file pone.0168262.s007.pdf]

S7 Table. Results of Seasonal Vaccination Analyses Reporting Relative Effects

Pandemic	Study	N Studies	Population Size (N)	Vaccine Type	Outcome	Risk Control Group	Risk With Intervention	Relative Effect (95% CI)
2009 H1N1	Li et al., 2015	4 RCTs	1,515	Seasonal influenza vaccine	Laboratory-confirmed infection	11/633 (17.4/1,000)	19/882 (21.5/1,000)	RR: 1.13 (0.56-2.29)
2009 H1N1	Li et al., 2015	16 case-control	40,868	Seasonal influenza vaccine	Laboratory-confirmed infection	8191/28,814 (284.3/1,000)	2,592/12,054 (215.0/1,000)	OR: 0.80 (0.61-1.05)
2009 H1N1	Yin et al., 2012	11 Case-control	31,699	Seasonal trivalent influenza vaccine	Laboratory confirmed influenza	6,599/21,907 (301.2/1,000)	2,282/9,792 (233.0/1,000)	OR: 0.81 (0.58-1.13)
2009 H1N1	Yin et al., 2012	6 (Excluded 5 studies with high risk of bias	28,292	Seasonal trivalent influenza vaccine	Laboratory confirmed influenza	5,851/19,613 (298.3/1,000)	1,827/8,679 (210.5/1,000)	OR: 0.66 (0.48-0.91)
