# Supplementary material for: Assessing the State of Knowledge Regarding the Effectiveness of Interventions to Contain Pandemic Influenza Transmission: A Systematic Review and Narrative Synthesis
Source: PLoS One. 2016 Dec 15;11(12):e0168262. doi: 10.1371/journal.pone.0168262 (PMC5158032; doi:10.1371/journal.pone.0168262)
Supplement: S8 Table — (PDF) [file pone.0168262.s008.pdf]

S8 Table. Results of Personal Protective Measure Analyses Reporting Relative Effects

Pandemic	Study	N Studies	Population Size (N)	Intervention	Outcome	Risk Control Group	Risk With Intervention	Relative Effect (95% CI)
2009 H1N1	Wong et al., 2014	1	149	Hand hygiene + mask use	Laboratory-confirmed influenza	19/82 (231.7/1,000)	10/67 (149.3/1,000)	0.64 (0.32-1.29)
2009 H1N1	Wong et al., 2014	1	149	Hand hygiene + mask use	ILI	14/82 (170.7/1,000)	6/67 (89.6/1,000)	0.52 (0.21-1.29)
