# Supplementary material for: Assessing the State of Knowledge Regarding the Effectiveness of Interventions to Contain Pandemic Influenza Transmission: A Systematic Review and Narrative Synthesis
Source: PLoS One. 2016 Dec 15;11(12):e0168262. doi: 10.1371/journal.pone.0168262 (PMC5158032; doi:10.1371/journal.pone.0168262)
Supplement: S9 Table — (PDF) [file pone.0168262.s009.pdf]

S9 Table. AMSTAR Scoring of Included Studies

Source	Criterion											Total	Category
	1	2	3	4	5	6	7	8	9	10	11		
Breteler et al., 2013	1	1	1	1	1	1	1	1	1	0	1	10	High
Chien et al., 2010	1	0	0	1	0	1	1	1	1	1	1	8	Moderate
Demicheli et al., 2014	1	1	1	1	1	1	1	1	1	1	1	11	High
Fielding et al., 2014	0	1	0	1	1	1	0	0	1	0	0	5	Moderate
Jackson et al., 2013	1	1	1	1	0	1	0	0	1	1	1	8	Moderate
Jefferson et al., 2008	1	1	1	1	0	1	1	1	1	0	1	9	High
Jefferson et al., 2014	1	1	1	1	1	1	1	1	1	1	1	11	High
Li et al., 2015	1	1	1	1	1	1	1	1	1	1	1	11	High
Li et al., 2016	1	1	1	1	0	1	1	1	1	0	1	9	High
Manzoli et al., 2011	0	1	1	1	0	0	1	1	1	1	1	8	Moderate
Mizumoto et al., 2013	1	0	0	0	1	1	0	0	1	0	1	5	Moderate
Mukerji et al., 2015	1	0	0	0	0	1	0	0	0	0	1	3	Low
Osterholm et al., 2012	1	0	0	1	0	1	0	0	1	0	1	5	Moderate
Perez Velasco et al., 2012	1	1	1	1	1	1	1	1	1	0	1	10	High
Wong et al., 2014	1	1	0	1	1	1	1	1	1	1	1	10	High
Yin et al., 2012	1	1	1	1	1	1	1	1	1	1	1	11	High
Yin et al., 2011	1	1	1	1	1	1	1	1	0	0	1	9	High
